# Supplementary material for: Automatic design of stigmergy-based behaviours for robot swarms
Source: Commun Eng. 2024 Feb 14;3:30. doi: 10.1038/s44172-024-00175-7 (PMC10956014; doi:10.1038/s44172-024-00175-7)
Supplement: Supplementary file 2 — supplementary information [file 44172_2024_175_MOESM2_ESM.pdf]

Supplementary information for the article: Automatic design of stigmergy-based behaviours for robot swarms

Muhammad Salman, David Garzón Ramos, and Mauro Birattari

Contents

1 Supplementary Note 1: Scalability of automatically generated stigmergy-based behaviours for a swarm of robots 5

2 Supplementary Note 2: Robustness to the reality gap 9

3 Supplementary Note 3: Manual control software design for robot swarms 11

4 Supplementary Note 4: Manual design spreadsheet 21

5 Supplementary Note 5: Technical datasheet 23

Chapter 1

Supplementary Note 1: Scalability of automatically generated stigmergy-based behaviours for a swarm of robots

In a robot swarm, the behaviour of each individual robot is affected by the actions of its neighbouring peer. It is therefore to be expected that the performance of the swarm might vary with the robot density, and therefore be affected by the number of robots and by the size of the environment. In this study, due to the limitations of the available experimental equipment, we were constrained to perform real-robot experiments with eight robots. Therefore, we explored the scalability of the stigmergy-based behaviours produced by `Habanero` through experiments in simulation. We considered nine scenarios characterised by different combinations of three arena sizes and three swarm sizes.

The three arena sizes are categorised as small ($1.7\,\mathrm{m}^2$), medium ($4.5\,\mathrm{m}^2$), and large ($12.0\,\mathrm{m}^2$). The ratio of the surface area of the small to the medium arena is similar the ratio of the medium to the large arena. The layout of the three arenas is shown in Fig. S1.1, while their simulation counterparts are shown in Fig. S1.2. For the Decision Making, Rendezvous Point, and Stop missions, we designated specific zones and obstacles according to the mission objectives. The three different swarm sizes consist of 8, 22, and 56 robots, respectively.

We conducted simulations to evaluate the instances of the control software produced by `Habanero` for each mission across the nine scenarios. We also evaluated the control software produced by `EvoPheromone`. The performance of the robot swarms is shown in Fig. S1.3. The results suggest a complex relationship involving the number of robots in the swarm, the size of the arena, and performance.

In Aggregation, swarm performance decreases with an increase in swarm size. This trend is consistent for both `Habanero` and `EvoPheromone`. The larger the swarm size, the greater the likelihood that local robot clusters form,, thereby increasing the average distance between robots and reducing the performance.

In Decision Making, performance also decreases with increasing swarm size. In a smaller arena, scoring zones are spatially constrained, unable to accommodate all robots, whereas, in medium and large arenas, robots require more time to locate the zone. It should be noted that once half the experiment's duration has passed, the zone indication lights are switched off, leaving the robots to depend on stigmergy to locate and remain in the correct zone.

Performance achieved in Rendezvous Point showed no significant improvement or decline for control software produced by both `Habanero` and `EvoPheromone`. In Stop, however, `Habanero` performed progressively better as swarm size increased. In contrast, `EvoPheromone`'s performance in a small arena rose with an increase in swarm size, but for the medium and the large arenas, performance rose initially with swarm size but followed by a decline with further increase in the swarm size.

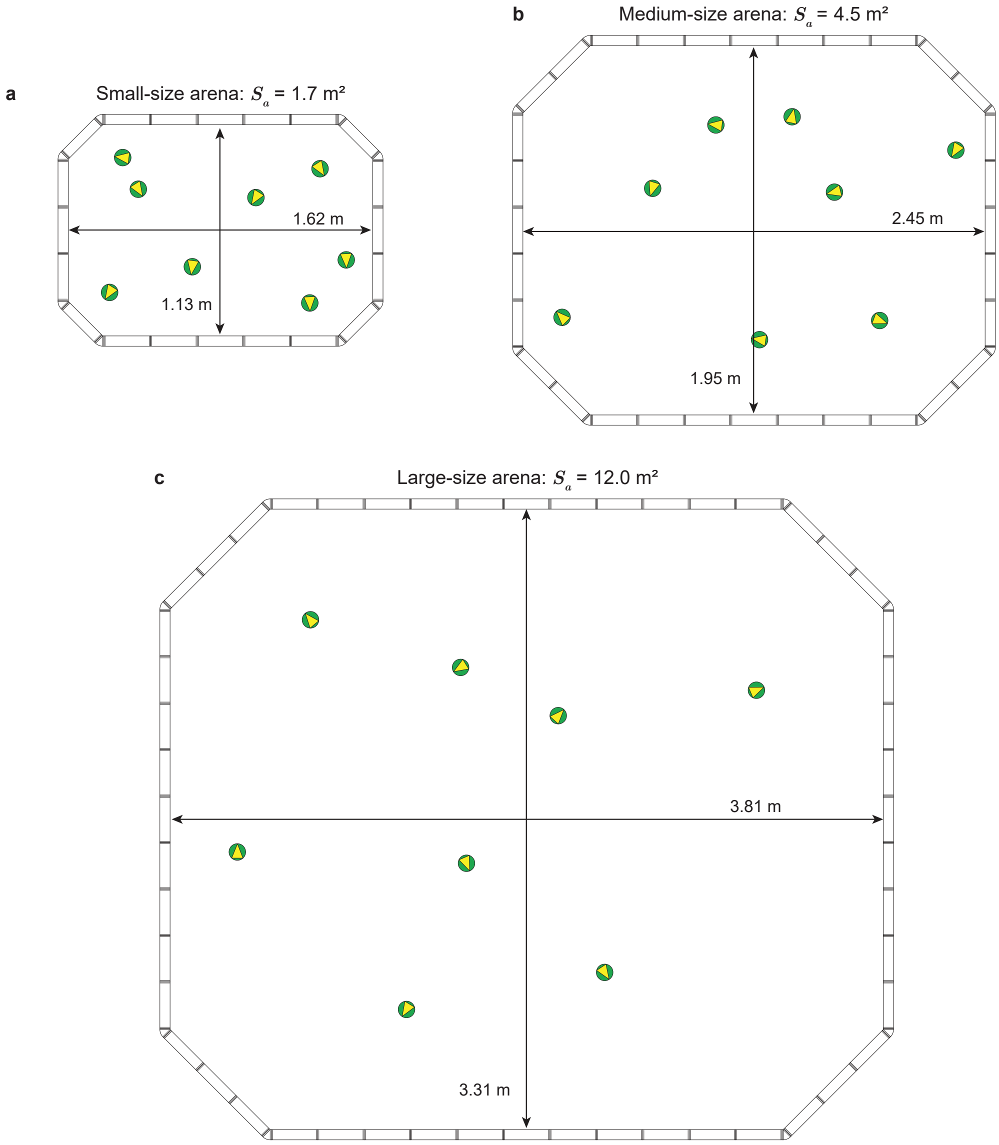

Figure S1.1: **Layout of the arenas for scalability experiments.** (**a**) Small-size arena. This arena is the one used in the real-robot experiments. (**b**) Medium-size arena. (**c**) Large-size arena.

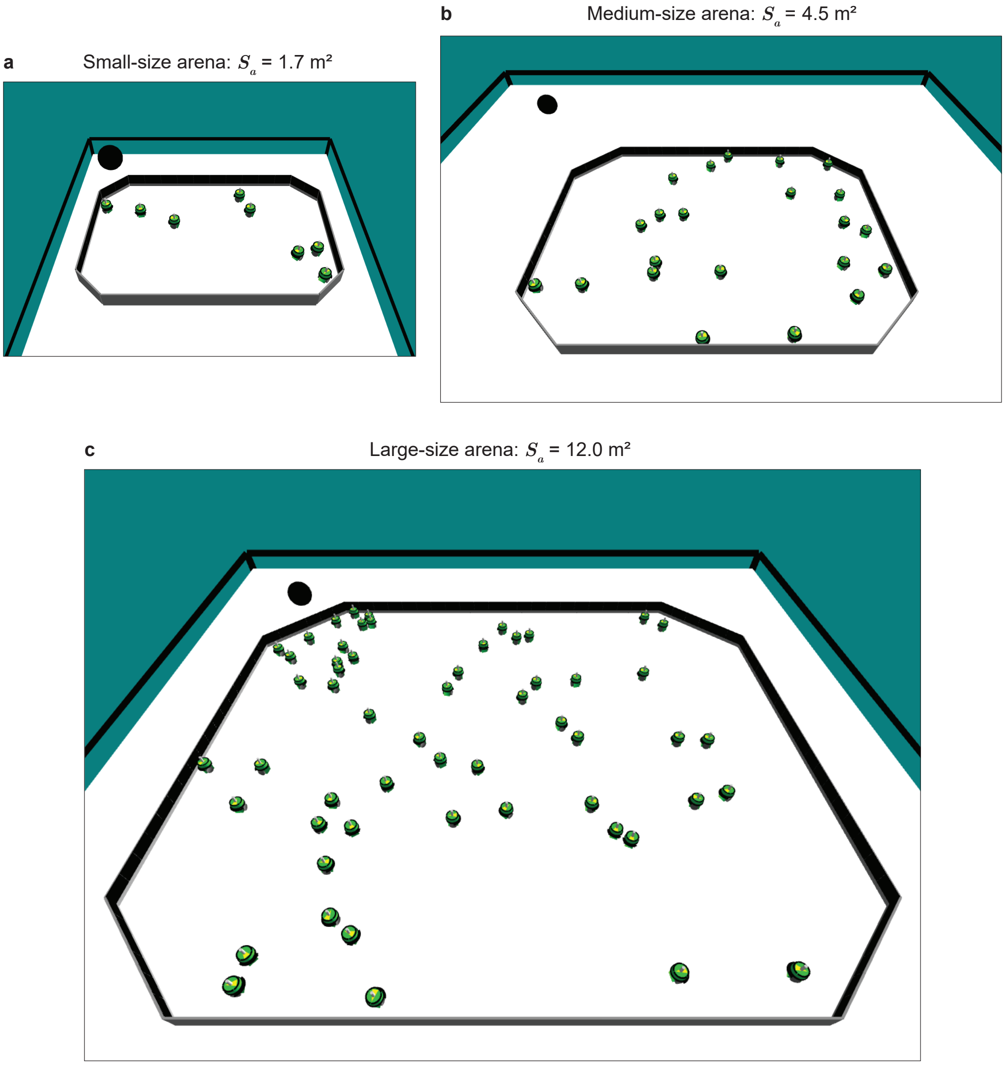

Figure S1.2: **Simulated arenas for scalability experiments.** (**a**) Small-size arena. (**b**) Medium-size arena. (**c**) Large-size arena.

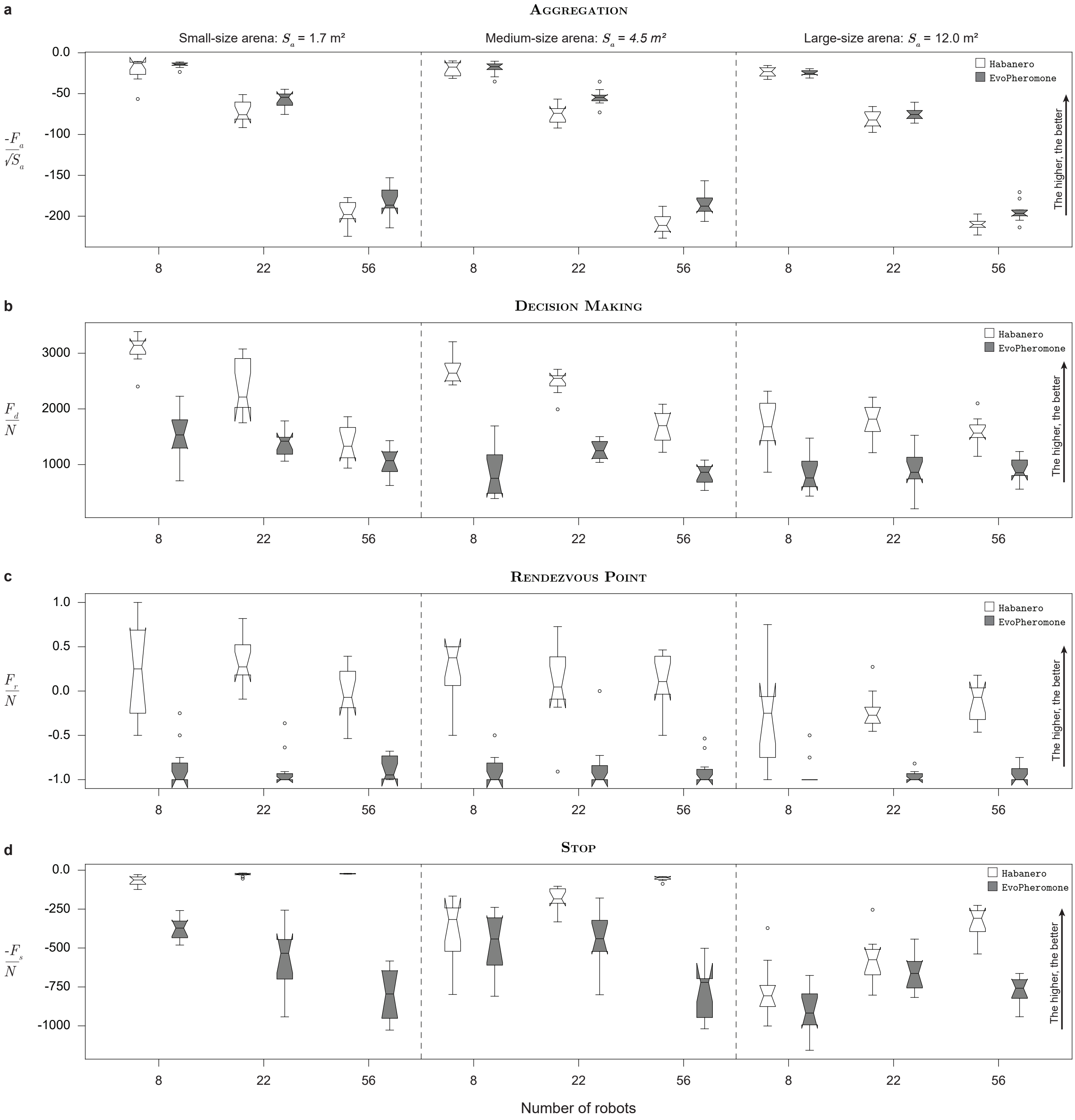

Figure S1.3: **Performance achieved by robot swarms in different scalability scenarios.** The results are presented using boxplots on a per-mission basis: (**a**) Aggregation, (**b**) Decision Making, (**c**) Rendezvous Point, and (**d**) Stop. The Aggregation performance is scaled by the square root of the size of the arena. The performance achieved in Decision Making, Rendezvous Point, and Stop are scaled by the number of robots in a swarm N.

Chapter 2

Supplementary Note 2: Robustness to the reality gap

In Fig. S2.1, we present the reality gap observed in the experiments conducted in this study. In both Aggregation and Rendezvous Point, `Habanero` demonstrates a smaller performance drop compared to `EvoPheromone`. In Stop, the performance drop is similar for `Habanero`, `EvoPheromone`, and `Human-Designers`. However, in Decision Making, the performance drop for `EvoPheromone` is less than that of `Habanero`. Yet, this is due to a sort of a floor effect: the performance of `EvoPheromone` was already particularly poor in simulation and this bounded its drop. All in all, although `Habanero` experienced a larger drop, it still outperformed `EvoPheromone`—see Fig. 5 in the manuscript.

The performance of `Random-Walk` remained largely consistent. This is because `Random-Walk` is not a design method, meaning there is no optimisation process involved and therefore overfitting does not happen.

We refer the reader to Francesca et. al [57], Ligot & Birattari [47], and Hasselmann et al. [48] for an in-depth discussion on the effects of the reality gap in modular and neuroevolutionary methods.

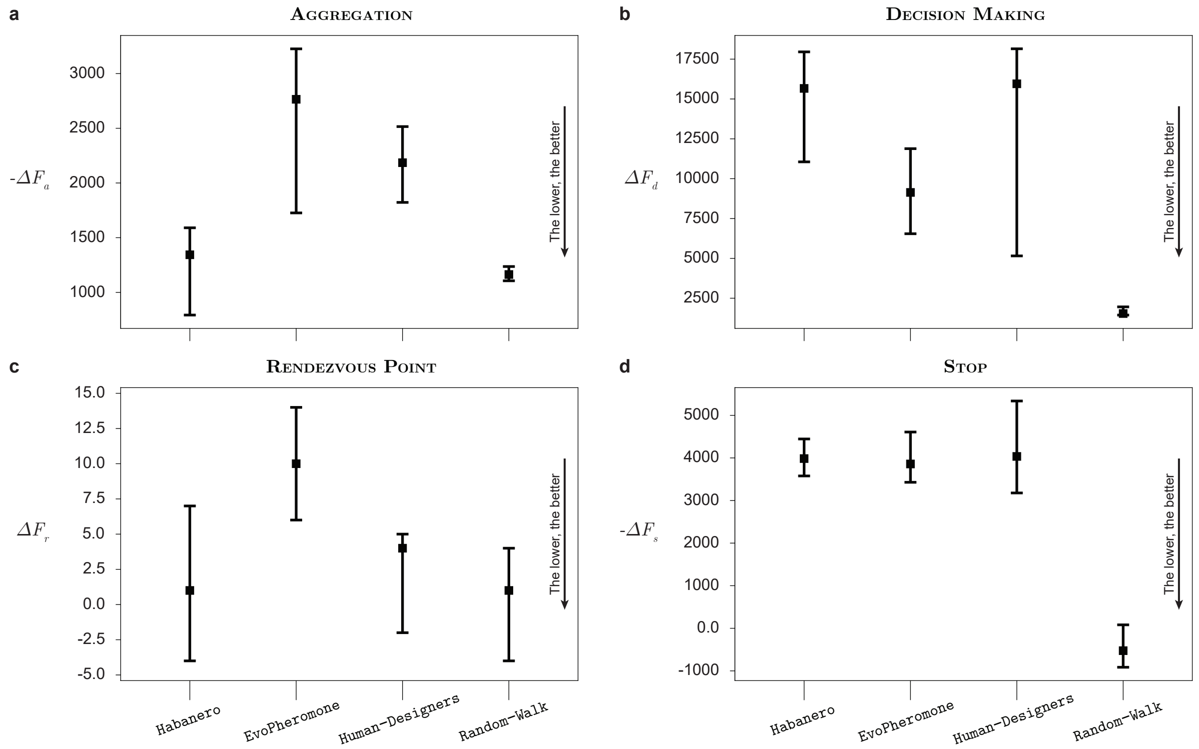

Figure S2.1: **Performance drop.** We present the performance drop using error bars on a per-mission basis: (**a**) AGGREGATION, (**b**) DECISION MAKING, (**c**) RENDEZVOUS POINT, and (**d**) STOP. Vertical segments represent the 95% confidence interval in the median, computed using the Wilcoxon's Signed Ranks statistics.

Chapter 3

Supplementary Note 3: Manual control software design for robot swarms

We sent the following document to all human designers. We are attaching it here in its original format with contact information anonymised.

Manual Control Software Design for Robot Swarms

Software Architecture

The control software has the form of a probabilistic finite-state machine. In this architecture, states (Nodes) represent low-level behaviors that the robots execute, and transition conditions (Edges) represent events that trigger the change from one behavior to another. Each low-level behavior and transition condition is a parametric software module. We have designed seven behaviors (nodes) and six transitions (edges) for this study. You will obtain the control software of the robots by assembling and configuring these software modules into a finite-state machines.

Software modules

Following table shows the description of the low level behaviors:

**Behaviors (Nodes)**	**Parameters**	**Description**
Exploration	*phe*	Robot moves by random walk.
Stop	phe	Robot stops at its place
Go-to-Color	*c, phe, fov*	Robot steadily moves toward objects displaying a specific color
Go-Away-Color	*c, phe, fov*	Robot steadily moves away from objects displaying a specific color.
Go-to-Pheromone	*phe, fov*	Robot steadily moves toward pheromone (Magenta color on the floor)
Avoid-Pheromone	*phe, fov*	Robot steadily moves away from pheromone (Magenta color on the floor).
Waggle	*phe*	Robot rotates in place for a random period of time.

- All low-level behaviors are capable of releasing pheromone ***phe*** *∈ {0, 1, 2}.* The width of a pheromone trail (magenta color) can be controlled by *phe.* For No-

pheromone, thin trail, or thick trail you can respectively select 0, 1, and 2 as values for phe.

- The parameter *fov* ∈ *{0, 1}* determines the field of view of the camera. *fov* = *0* means the camera can only see in front of it (directional view) and *fov* = *1* means camera has an omni vision.
- The parameter *c* ∈ *{R, G, B, Y, C},* determines the color to be Go-toward or move away
  - c = 1 -> Red
  - c = 2 -> Green
  - c = 3 -> Blue
  - c = 4 -> Yellow
  - c = 5 -> Cyan

Following table shows the description of the condition transitions:

**Transitions (Edges)**	**Parameters**	**Description**
White-Floor	β	White floor detected
Gray-Floor	β	Gray floor detected
Black-Floor	β	Black floor detected
Color-Detected	β, c, fov	Objects of color perceived.
Pheromone-Detected	β, fov	Pheromone perceived.
Fixed-Probability	β	Transition with a fixed probability

The parameter $\beta \in [0, 1]$ determines the probability of transitioning.

A typical FSM for a robot swarm to perform Coverage (explore maximum area of an arena) is shown as Figure 1. The initial state (Exploration) is represented by a dark circle. The robots start Exploration with a thin Pheromone trail (*phe* = *1*). With a probability β = *0.1*, the robots switch their behavior from Exploration to Avoid-color. During Avoid-color, the robots perceive their surrounding with a field-of-view of 360-degree (*fov* = *1*) and avoid Red color (*c* = *1*) objects while dropping pheromone (*phe* = *1*). The robots again switch back to Exploration behavior with a probability of β = *0.1.* The robots keep executing this FSM till the end of the mission time.

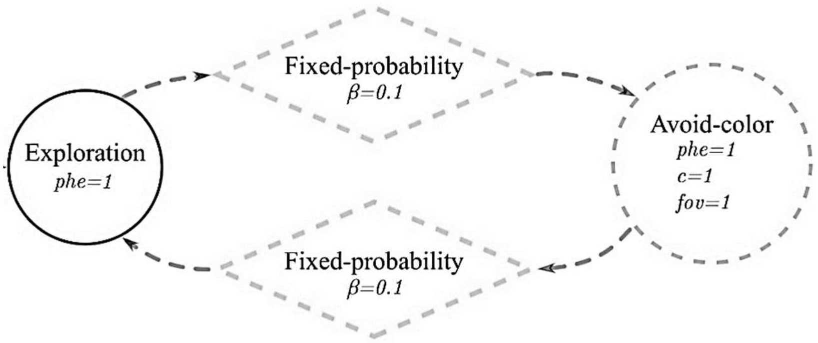

Figure 1: An example of a typical FSM

Collective Missions

You will design manual controllers for a robot swarm for four collective missions. In each mission, eight robots collaborate to accomplish a task. The maximum duration of a mission is 180 seconds and each second has 10 control steps.

1. Aggregation

At the beginning, the robots are randomly placed in the arena. The robots must get close to one another and remain in that formation till the end of the mission. At each control step, the average distance between the robots will be added to the score. We want to minimize the score.

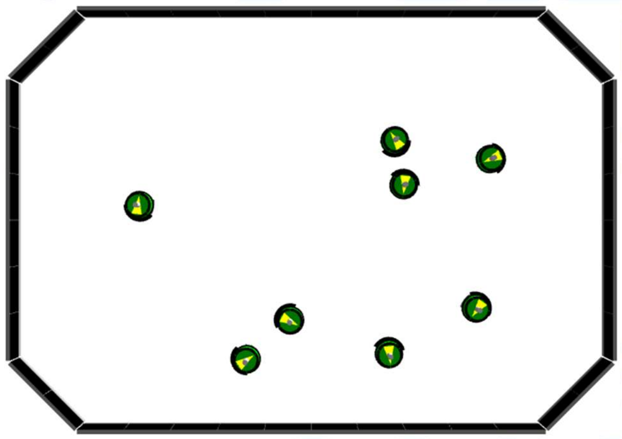

2. Decision Making

At the beginning, the robots are randomly placed in the arena. At every control step, if a robot is in the Green region, the score will increase by +1 and if a robot is in a Blue region, the score will increase by +2. Both Green and Blue light signals will disappear randomly between 70 to 90 seconds. The robots must gather maximum score at the end of the mission. The scores will be given in negative

values, however, the absolute value of the score will correspond to the time the robots spend either in the Green or Blue region.

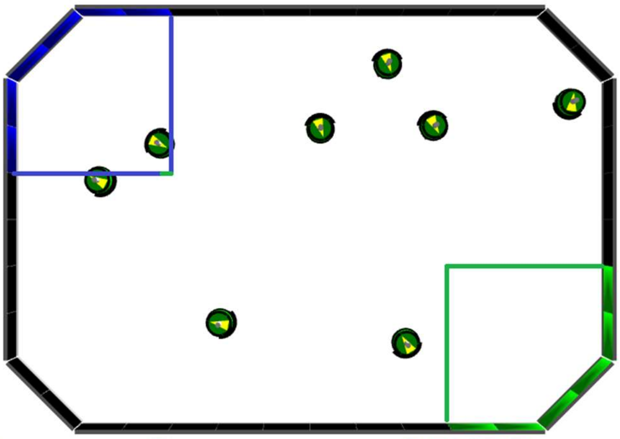

3. **Rendezvous Point**

At the beginning, the robots are randomly placed at the left side of the arena. The robots must reach at green region and stay there till the end of the mission. Both Green and Blue light signals will disappear randomly between 70 to 90 seconds. At the end of the mission, the objected function will be computed as (the number of robots inside green region) - (The number of robots outside the green region). We want to maximize the number of robots inside the green region at the end of the mission. The scores will be given in negative values, however, the absolute value of the score will correspond to the number of robots in the region. E.g., -6 is a better score than -3.

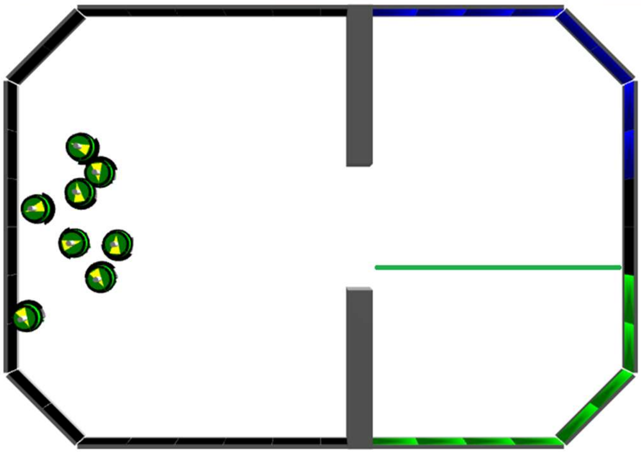

4. **Stop**

At the beginning, the robots are randomly placed in the arena. A Blue light signal will appear randomly between 70 to 90 seconds on a random block. All the robots must stop as soon as the signal appears. The score increases if robots stop before

Blue signal and Score also increases if robots do not stop after the signal. We want to minimize the score in this mission.

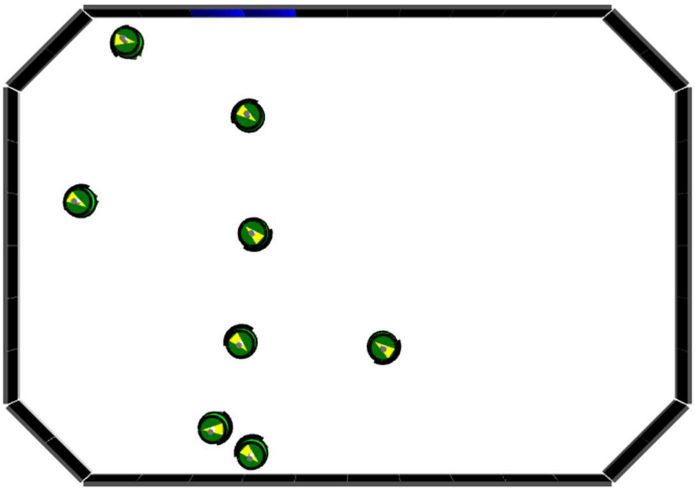

Protocol

- You can choose maximum four Behaviors (Nodes) to design an FSM.
- For each mission, you can only take maximum four hours to design a suitable FSM.
- There is no limit of minimum time. You can finish when you are satisfied with your design.

Instructions

We have setup six computers that are installed with a visualization tool. The visualization tool will allow you to easily drag and drop nodes and edges to create an FSM and execute the FSM to see the simulation of the robot swarm executing your FSM. You can modify/fine-tune your designed as much as possible.

Computer Access

With the following link to an online spreadsheet, you can choose any free time-slot on any free computer.

Online-Spread-sheet *(this spread sheet is provided in instructions-for-designers directory)*

If you want to reserve a time-slot or you are facing any issue with the connectivity or other technical issue, please immediately contact Anonymized

Whattsapp: Anonymized

Phone: Anonymized
Email: Anonymized

Launching Visualization Tool

Once you are connected to a PC, open the terminal. Launch a mission by one of the following steps (You can only launch one mission at a time).

1. To launch Visualization Tool for Aggregation mission, enter

```
./start_e1_aggregation.sh
```

2. To launch Visualization Tool for Decision Making mission, enter

```
./start_e2_decision.sh
```

3. To launch Visualization Tool for Rendezvous Point mission, enter

```
./start_e3_rendezvous.sh
```

4. To launch Visualization Tool for Stop mission, enter

```
./start_e4_stop.sh
```

Once you enter a command; the visualization tool will appear in Firefox browser.

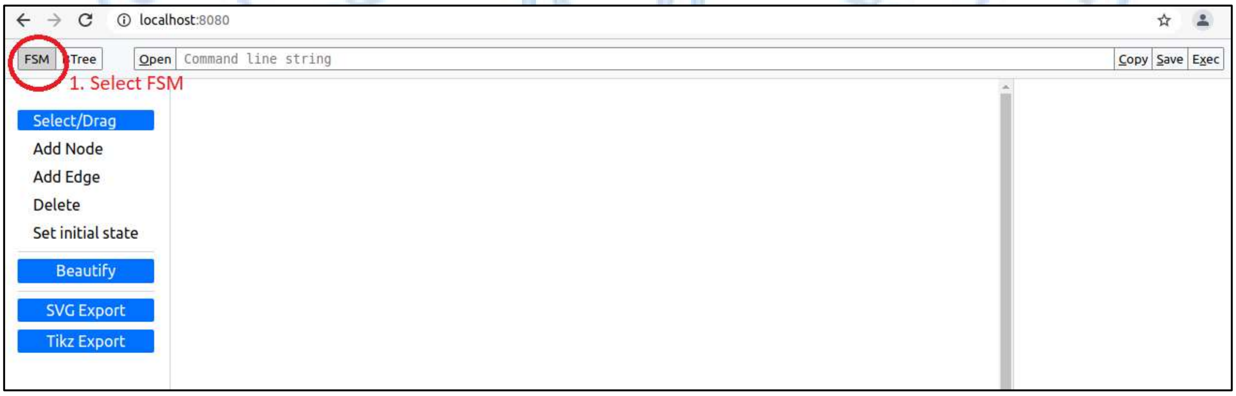

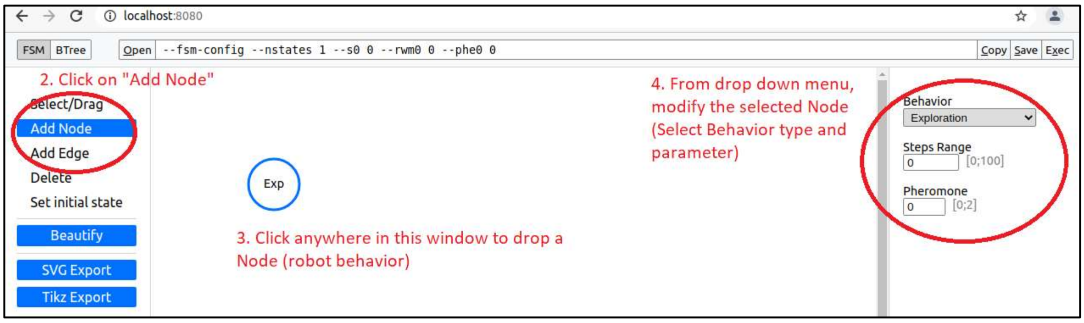

You can place maximum four nodes. Once you have placed your desired nodes, you will click on "Add Edge". To connect an Edge between two nodes, you will first click on the node from where the Edge is starting, and you will do the second click on the node where the Edge is terminating. For instance, as shown below, to place a Black-floor transition (Edge) between Exploration (Exp) and Stop, you will first click on the Exp and then on the Stop.

Once you are ready to test your design, click on "Exec" to execute the simulation.

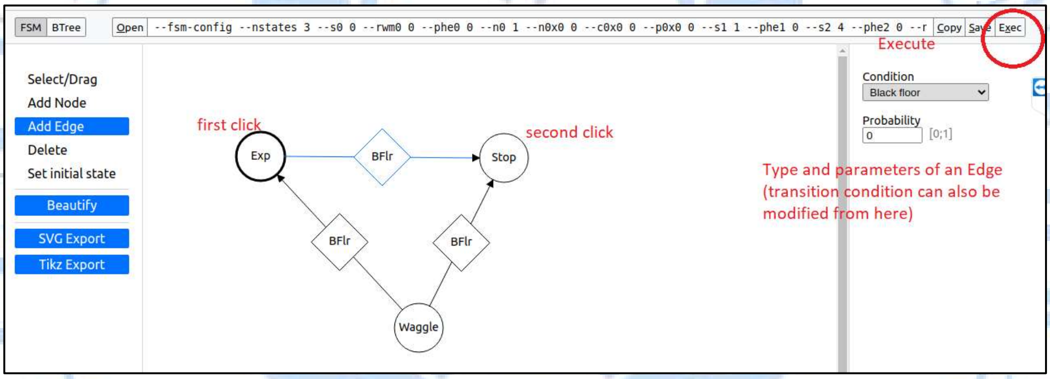

A simulation window will appear. Initially the robots might appear outside of the arena. It is a visualization glitch and does not affect anything. You can observe the behavior of the robots and the score in the simulation.

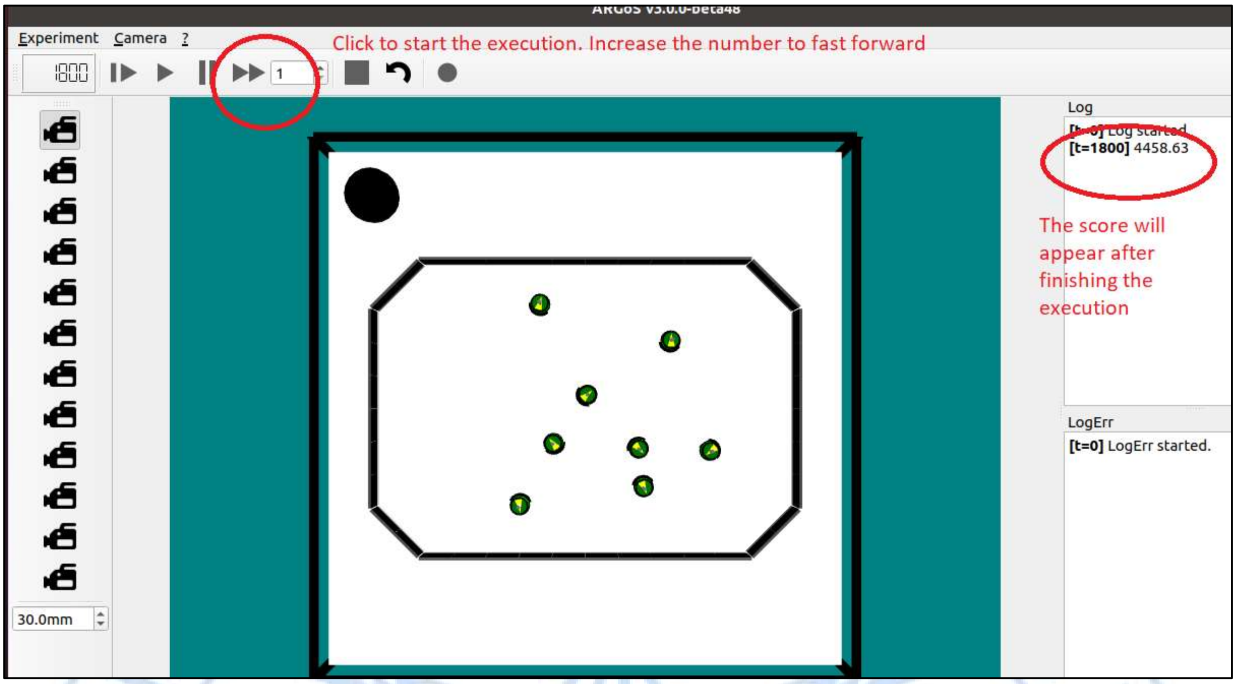

Next, you will close this simulation window and go back to the visualization tool. If you want to modify your design, you can do it as many times as you want. When you are satisfied with the results. Save the design by clicking "Save". A file will download. You have to rename it and save it at an appropriate location.

You will generate one such file for each mission and send us those files.

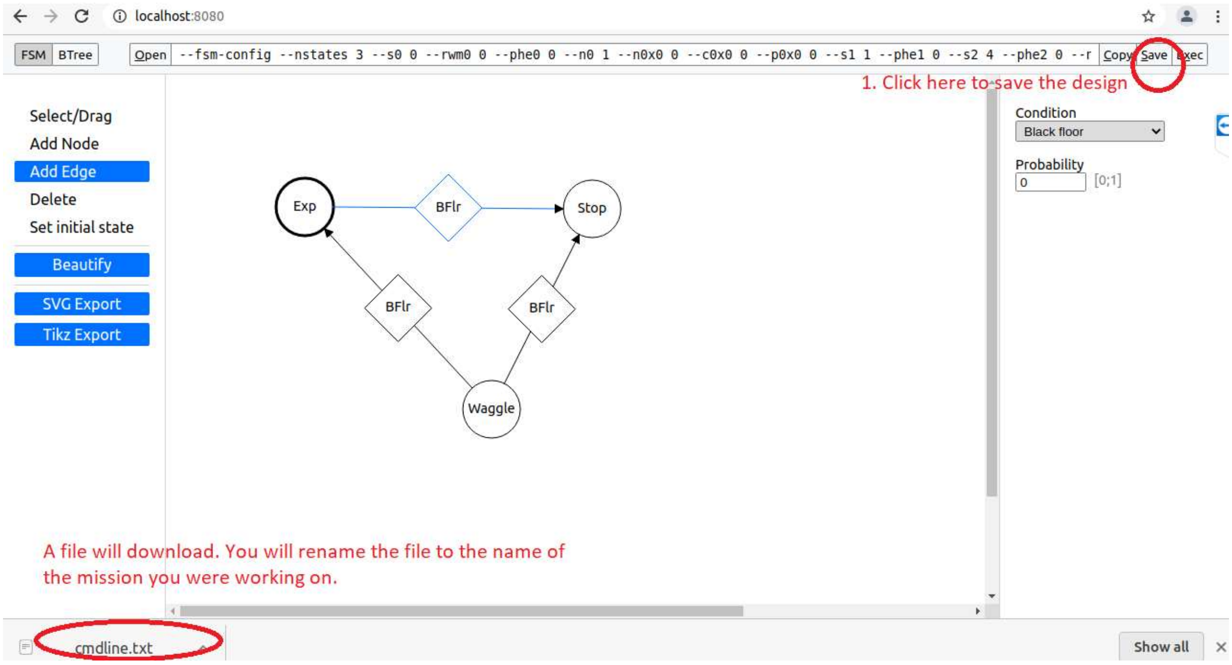

Now you have finished one mission. In order to go for the next mission; Close the Firefox browser; and enter one of the following commands to launch the next mission.

1. To launch Visualization Tool for Aggregation mission, enter

```
./start_e1_aggregation.sh
```

2. To launch Visualization Tool for Decision Making mission, enter

```
./start_e2_decision.sh
```

3. To launch Visualization Tool for Rendezvous Point mission, enter

```
./start_e3_rendezvous.sh
```

4. To launch Visualization Tool for Stop mission, enter

```
./start_e4_stop.sh
```

You will repeat these steps for all four missions.

Chapter 4

Supplementary Note 4: Manual design spreadsheet

	1-May-2021		2-May-2021		3-May-2021		4-May-2021		5-May-2021		6-May-2021		7-May-2021		8-May-2021		9-May-2021	
	SATURDAY		SUNDAY		MONDAY		TUESDAY		WEDNESDAY		THURSDAY		FRIDAY		SATURDAY		SUNDAY	
**Computer-1**	1 AM - 5 AM	6 AM - 10 AM	1 AM - 5 AM	6 AM - 10 AM	1 AM - 5 AM	6 AM - 10 AM	1 AM - 5 AM	6 AM - 10 AM	1 AM - 5 AM	6 AM - 10 AM	1 AM - 5 AM	6 AM - 10 AM	1 AM - 5 AM	6 AM - 10 AM	1 AM - 5 AM	6 AM - 10 AM	1 AM - 5 AM	6 AM - 10 AM
Computer password: anonymized	5 AM - 9 AM	10 AM - 2 PM	5 AM - 9 AM	10 AM - 2 PM	5 AM - 9 AM	10 AM - 2 PM	5 AM - 9 AM	10 AM - 2 PM	5 AM - 9 AM	10 AM - 2 PM	5 AM - 9 AM	10 AM - 2 PM	5 AM - 9 AM	10 AM - 2 PM	5 AM - 9 AM	10 AM - 2 PM	5 AM - 9 AM	10 AM - 2 PM
Team viewer ID: anonymized	9 AM - 1 PM	2 PM - 6 PM	9 AM - 1 PM	2 PM - 6 PM	9 AM - 1 PM	2 PM - 6 PM	9 AM - 1 PM	2 PM - 6 PM	9 AM - 1 PM	2 PM - 6 PM	9 AM - 1 PM	2 PM - 6 PM	9 AM - 1 PM	2 PM - 6 PM	9 AM - 1 PM	2 PM - 6 PM	9 AM - 1 PM	2 PM - 6 PM
Team Viewer Password: anonymized	1 PM - 5 PM	6 PM - 10 PM	1 PM - 5 PM	6 PM - 10 PM	1 PM - 5 PM	6 PM - 10 PM	1 PM - 5 PM	6 PM - 10 PM	1 PM - 5 PM	6 PM - 10 PM	1 PM - 5 PM	6 PM - 10 PM	1 PM - 5 PM	6 PM - 10 PM	1 PM - 5 PM	6 PM - 10 PM	1 PM - 5 PM	6 PM - 10 PM
	5 PM - 9 PM	10 PM - 2 AM	5 PM - 9 PM	10 PM - 2 AM	5 PM - 9 PM	10 PM - 2 AM	5 PM - 9 PM	10 PM - 2 AM	5 PM - 9 PM	10 PM - 2 AM	5 PM - 9 PM	10 PM - 2 AM	5 PM - 9 PM	10 PM - 2 AM	5 PM - 9 PM	10 PM - 2 AM	5 PM - 9 PM	10 PM - 2 AM
	9 PM - 1 AM	2 AM - 6 AM	9 PM - 1 AM	2 AM - 6 AM	9 PM - 1 AM	2 AM - 6 AM	9 PM - 1 AM	2 AM - 6 AM	9 PM - 1 AM	2 AM - 6 AM	9 PM - 1 AM	2 AM - 6 AM	9 PM - 1 AM	2 AM - 6 AM	9 PM - 1 AM	2 AM - 6 AM	9 PM - 1 AM	2 AM - 6 AM
**Computer-2**	1 AM - 5 AM	6 AM - 10 AM	1 AM - 5 AM	6 AM - 10 AM	1 AM - 5 AM	6 AM - 10 AM	1 AM - 5 AM	6 AM - 10 AM	1 AM - 5 AM	6 AM - 10 AM	1 AM - 5 AM	6 AM - 10 AM	1 AM - 5 AM	6 AM - 10 AM	1 AM - 5 AM	6 AM - 10 AM	1 AM - 5 AM	6 AM - 10 AM
Computer password: anonymized	5 AM - 9 AM	10 AM - 2 PM	5 AM - 9 AM	10 AM - 2 PM	5 AM - 9 AM	10 AM - 2 PM	5 AM - 9 AM	10 AM - 2 PM	5 AM - 9 AM	10 AM - 2 PM	5 AM - 9 AM	10 AM - 2 PM	5 AM - 9 AM	10 AM - 2 PM	5 AM - 9 AM	10 AM - 2 PM	5 AM - 9 AM	10 AM - 2 PM
Team viewer ID: anonymized	9 AM - 1 PM	2 PM - 6 PM	9 AM - 1 PM	2 PM - 6 PM	9 AM - 1 PM	2 PM - 6 PM	9 AM - 1 PM	2 PM - 6 PM	9 AM - 1 PM	2 PM - 6 PM	9 AM - 1 PM	2 PM - 6 PM	9 AM - 1 PM	2 PM - 6 PM	9 AM - 1 PM	2 PM - 6 PM	9 AM - 1 PM	2 PM - 6 PM
Team Viewer Password:anonymized	1 PM - 5 PM	6 PM - 10 PM	1 PM - 5 PM	6 PM - 10 PM	1 PM - 5 PM	6 PM - 10 PM	1 PM - 5 PM	6 PM - 10 PM	1 PM - 5 PM	6 PM - 10 PM	1 PM - 5 PM	6 PM - 10 PM	1 PM - 5 PM	6 PM - 10 PM	1 PM - 5 PM	6 PM - 10 PM	1 PM - 5 PM	6 PM - 10 PM
	5 PM - 9 PM	10 PM - 2 AM	5 PM - 9 PM	10 PM - 2 AM	5 PM - 9 PM	10 PM - 2 AM	5 PM - 9 PM	10 PM - 2 AM	5 PM - 9 PM	10 PM - 2 AM	5 PM - 9 PM	10 PM - 2 AM	5 PM - 9 PM	10 PM - 2 AM	5 PM - 9 PM	10 PM - 2 AM	5 PM - 9 PM	10 PM - 2 AM
	9 PM - 1 AM	2 AM - 6 AM	9 PM - 1 AM	2 AM - 6 AM	9 PM - 1 AM	2 AM - 6 AM	9 PM - 1 AM	2 AM - 6 AM	9 PM - 1 AM	2 AM - 6 AM	9 PM - 1 AM	2 AM - 6 AM	9 PM - 1 AM	2 AM - 6 AM	9 PM - 1 AM	2 AM - 6 AM	9 PM - 1 AM	2 AM - 6 AM
**Computer-3**	1 AM - 5 AM	6 AM - 10 AM	1 AM - 5 AM	6 AM - 10 AM	1 AM - 5 AM	6 AM - 10 AM	1 AM - 5 AM	6 AM - 10 AM	1 AM - 5 AM	6 AM - 10 AM	1 AM - 5 AM	6 AM - 10 AM	1 AM - 5 AM	6 AM - 10 AM	1 AM - 5 AM	6 AM - 10 AM	1 AM - 5 AM	6 AM - 10 AM
Computer password: anonymized	5 AM - 9 AM	10 AM - 2 PM	5 AM - 9 AM	10 AM - 2 PM	5 AM - 9 AM	10 AM - 2 PM	5 AM - 9 AM	10 AM - 2 PM	5 AM - 9 AM	10 AM - 2 PM	5 AM - 9 AM	10 AM - 2 PM	5 AM - 9 AM	10 AM - 2 PM	5 AM - 9 AM	10 AM - 2 PM	5 AM - 9 AM	10 AM - 2 PM
Team viewer ID: anonymized	9 AM - 1 PM	2 PM - 6 PM	9 AM - 1 PM	2 PM - 6 PM	9 AM - 1 PM	2 PM - 6 PM	9 AM - 1 PM	2 PM - 6 PM	9 AM - 1 PM	2 PM - 6 PM	9 AM - 1 PM	2 PM - 6 PM	9 AM - 1 PM	2 PM - 6 PM	9 AM - 1 PM	2 PM - 6 PM	9 AM - 1 PM	2 PM - 6 PM
Team Viewer Password: anonymized	1 PM - 5 PM	6 PM - 10 PM	1 PM - 5 PM	6 PM - 10 PM	1 PM - 5 PM	6 PM - 10 PM	1 PM - 5 PM	6 PM - 10 PM	1 PM - 5 PM	6 PM - 10 PM	1 PM - 5 PM	6 PM - 10 PM	1 PM - 5 PM	6 PM - 10 PM	1 PM - 5 PM	6 PM - 10 PM	1 PM - 5 PM	6 PM - 10 PM
	5 PM - 9 PM	10 PM - 2 AM	5 PM - 9 PM	10 PM - 2 AM	5 PM - 9 PM	10 PM - 2 AM	5 PM - 9 PM	10 PM - 2 AM	5 PM - 9 PM	10 PM - 2 AM	5 PM - 9 PM	10 PM - 2 AM	5 PM - 9 PM	10 PM - 2 AM	5 PM - 9 PM	10 PM - 2 AM	5 PM - 9 PM	10 PM - 2 AM
	9 PM - 1 AM	2 AM - 6 AM	9 PM - 1 AM	2 AM - 6 AM	9 PM - 1 AM	2 AM - 6 AM	9 PM - 1 AM	2 AM - 6 AM	9 PM - 1 AM	2 AM - 6 AM	9 PM - 1 AM	2 AM - 6 AM	9 PM - 1 AM	2 AM - 6 AM	9 PM - 1 AM	2 AM - 6 AM	9 PM - 1 AM	2 AM - 6 AM
**Computer-4**	1 AM - 5 AM	6 AM - 10 AM	1 AM - 5 AM	6 AM - 10 AM	1 AM - 5 AM	6 AM - 10 AM	1 AM - 5 AM	6 AM - 10 AM	1 AM - 5 AM	6 AM - 10 AM	1 AM - 5 AM	6 AM - 10 AM	1 AM - 5 AM	6 AM - 10 AM	1 AM - 5 AM	6 AM - 10 AM	1 AM - 5 AM	6 AM - 10 AM
Computer password: anonymized	5 AM - 9 AM	10 AM - 2 PM	5 AM - 9 AM	10 AM - 2 PM	5 AM - 9 AM	10 AM - 2 PM	5 AM - 9 AM	10 AM - 2 PM	5 AM - 9 AM	10 AM - 2 PM	5 AM - 9 AM	10 AM - 2 PM	5 AM - 9 AM	10 AM - 2 PM	5 AM - 9 AM	10 AM - 2 PM	5 AM - 9 AM	10 AM - 2 PM
Team viewer ID: anonymized	9 AM - 1 PM	2 PM - 6 PM	9 AM - 1 PM	2 PM - 6 PM	9 AM - 1 PM	2 PM - 6 PM	9 AM - 1 PM	2 PM - 6 PM	9 AM - 1 PM	2 PM - 6 PM	9 AM - 1 PM	2 PM - 6 PM	9 AM - 1 PM	2 PM - 6 PM	9 AM - 1 PM	2 PM - 6 PM	9 AM - 1 PM	2 PM - 6 PM
Team Viewer Password: anonymized	1 PM - 5 PM	6 PM - 10 PM	1 PM - 5 PM	6 PM - 10 PM	1 PM - 5 PM	6 PM - 10 PM	1 PM - 5 PM	6 PM - 10 PM	1 PM - 5 PM	6 PM - 10 PM	1 PM - 5 PM	6 PM - 10 PM	1 PM - 5 PM	6 PM - 10 PM	1 PM - 5 PM	6 PM - 10 PM	1 PM - 5 PM	6 PM - 10 PM
	5 PM - 9 PM	10 PM - 2 AM	5 PM - 9 PM	10 PM - 2 AM	5 PM - 9 PM	10 PM - 2 AM	5 PM - 9 PM	10 PM - 2 AM	5 PM - 9 PM	10 PM - 2 AM	5 PM - 9 PM	10 PM - 2 AM	5 PM - 9 PM	10 PM - 2 AM	5 PM - 9 PM	10 PM - 2 AM	5 PM - 9 PM	10 PM - 2 AM
	9 PM - 1 AM	2 AM - 6 AM	9 PM - 1 AM	2 AM - 6 AM	9 PM - 1 AM	2 AM - 6 AM	9 PM - 1 AM	2 AM - 6 AM	9 PM - 1 AM	2 AM - 6 AM	9 PM - 1 AM	2 AM - 6 AM	9 PM - 1 AM	2 AM - 6 AM	9 PM - 1 AM	2 AM - 6 AM	9 PM - 1 AM	2 AM - 6 AM
**TrackingSystem-5**	1 AM - 5 AM	6 AM - 10 AM	1 AM - 5 AM	6 AM - 10 AM	1 AM - 5 AM	6 AM - 10 AM	1 AM - 5 AM	6 AM - 10 AM	1 AM - 5 AM	6 AM - 10 AM	1 AM - 5 AM	6 AM - 10 AM	1 AM - 5 AM	6 AM - 10 AM	1 AM - 5 AM	6 AM - 10 AM	1 AM - 5 AM	6 AM - 10 AM
Computer password: anonymized	5 AM - 9 AM	10 AM - 2 PM	5 AM - 9 AM	10 AM - 2 PM	5 AM - 9 AM	10 AM - 2 PM	5 AM - 9 AM	10 AM - 2 PM	5 AM - 9 AM	10 AM - 2 PM	5 AM - 9 AM	10 AM - 2 PM	5 AM - 9 AM	10 AM - 2 PM	5 AM - 9 AM	10 AM - 2 PM	5 AM - 9 AM	10 AM - 2 PM
Team viewer ID: anonymized	9 AM - 1 PM	2 PM - 6 PM	9 AM - 1 PM	2 PM - 6 PM	9 AM - 1 PM	2 PM - 6 PM	9 AM - 1 PM	2 PM - 6 PM	9 AM - 1 PM	2 PM - 6 PM	9 AM - 1 PM	2 PM - 6 PM	9 AM - 1 PM	2 PM - 6 PM	9 AM - 1 PM	2 PM - 6 PM	9 AM - 1 PM	2 PM - 6 PM
Team Viewer Password: anonymized	1 PM - 5 PM	6 PM - 10 PM	1 PM - 5 PM	6 PM - 10 PM	1 PM - 5 PM	6 PM - 10 PM	1 PM - 5 PM	6 PM - 10 PM	1 PM - 5 PM	6 PM - 10 PM	1 PM - 5 PM	6 PM - 10 PM	1 PM - 5 PM	6 PM - 10 PM	1 PM - 5 PM	6 PM - 10 PM	1 PM - 5 PM	6 PM - 10 PM
	5 PM - 9 PM	10 PM - 2 AM	5 PM - 9 PM	10 PM - 2 AM	5 PM - 9 PM	10 PM - 2 AM	5 PM - 9 PM	10 PM - 2 AM	5 PM - 9 PM	10 PM - 2 AM	5 PM - 9 PM	10 PM - 2 AM	5 PM - 9 PM	10 PM - 2 AM	5 PM - 9 PM	10 PM - 2 AM	5 PM - 9 PM	10 PM - 2 AM
	9 PM - 1 AM	2 AM - 6 AM	9 PM - 1 AM	2 AM - 6 AM	9 PM - 1 AM	2 AM - 6 AM	9 PM - 1 AM	2 AM - 6 AM	9 PM - 1 AM	2 AM - 6 AM	9 PM - 1 AM	2 AM - 6 AM	9 PM - 1 AM	2 AM - 6 AM	9 PM - 1 AM	2 AM - 6 AM	9 PM - 1 AM	2 AM - 6 AM
**Masters-6**	1 AM - 5 AM	6 AM - 10 AM	1 AM - 5 AM	6 AM - 10 AM	1 AM - 5 AM	6 AM - 10 AM	1 AM - 5 AM	6 AM - 10 AM	1 AM - 5 AM	6 AM - 10 AM	1 AM - 5 AM	6 AM - 10 AM	1 AM - 5 AM	6 AM - 10 AM	1 AM - 5 AM	6 AM - 10 AM	1 AM - 5 AM	6 AM - 10 AM
Computer password: anonymized	5 AM - 9 AM	10 AM - 2 PM	5 AM - 9 AM	10 AM - 2 PM	5 AM - 9 AM	10 AM - 2 PM	5 AM - 9 AM	10 AM - 2 PM	5 AM - 9 AM	10 AM - 2 PM	5 AM - 9 AM	10 AM - 2 PM	5 AM - 9 AM	10 AM - 2 PM	5 AM - 9 AM	10 AM - 2 PM	5 AM - 9 AM	10 AM - 2 PM
Team viewer ID: anonymized	9 AM - 1 PM	2 PM - 6 PM	9 AM - 1 PM	2 PM - 6 PM	9 AM - 1 PM	2 PM - 6 PM	9 AM - 1 PM	2 PM - 6 PM	9 AM - 1 PM	2 PM - 6 PM	9 AM - 1 PM	2 PM - 6 PM	9 AM - 1 PM	2 PM - 6 PM	9 AM - 1 PM	2 PM - 6 PM	9 AM - 1 PM	2 PM - 6 PM
Team Viewer Password: anonymized	1 PM - 5 PM	6 PM - 10 PM	1 PM - 5 PM	6 PM - 10 PM	1 PM - 5 PM	6 PM - 10 PM	1 PM - 5 PM	6 PM - 10 PM	1 PM - 5 PM	6 PM - 10 PM	1 PM - 5 PM	6 PM - 10 PM	1 PM - 5 PM	6 PM - 10 PM	1 PM - 5 PM	6 PM - 10 PM	1 PM - 5 PM	6 PM - 10 PM
	5 PM - 9 PM	10 PM - 2 AM	5 PM - 9 PM	10 PM - 2 AM	5 PM - 9 PM	10 PM - 2 AM	5 PM - 9 PM	10 PM - 2 AM	5 PM - 9 PM	10 PM - 2 AM	5 PM - 9 PM	10 PM - 2 AM	5 PM - 9 PM	10 PM - 2 AM	5 PM - 9 PM	10 PM - 2 AM	5 PM - 9 PM	10 PM - 2 AM
	9 PM - 1 AM	2 AM - 6 AM	9 PM - 1 AM	2 AM - 6 AM	9 PM - 1 AM	2 AM - 6 AM	9 PM - 1 AM	2 AM - 6 AM	9 PM - 1 AM	2 AM - 6 AM	9 PM - 1 AM	2 AM - 6 AM	9 PM - 1 AM	2 AM - 6 AM	9 PM - 1 AM	2 AM - 6 AM	9 PM - 1 AM	2 AM - 6 AM

	NAME	Color	Documentation	Status	Notes	Experience
1	Designer 1 (anonymized)		Sent	Completed	Collected	Postdoc in swarm robotics
2	Designer 2 (anonymized)		Sent	Completed	Collected	Master in swarm robotics
3	Designer 3 (anonymized)		Sent	Completed	Collected	PhD swarm robotics
4	Designer 4 (anonymized)		Sent	Completed	Collected	Master in swarm robotics (ongoing)
5	Designer 5 (anonymized)		Sent	Completed	Collected	MA1 in swarm robotics
6	Designer 6 (anonymized)		Sent	in-progress (1 mission)	Stopped	
7	Designer 7 (anonymized)		Sent	Completed	Collected	Master in swarm robotics (ongoing)
8	Designer 8 (anonymized)		Sent	Completed	Collected	PhD swarm robotics (ongoing)
9	Designer 9 (anonymized)		Sent	Completed	Collected	BA4 in swarm robotics (ongoing)
10	Designer 10 (anonymized)				Not available	
11	Designer 11 (anonymized)		Sent	Not started	Stopped	
12	Designer 12 (anonymized)		Sent	Completed	Collected	Prof. research in swarm robotics
13	Designer 13 (anonymized)				Writting email	
14	Designer 14 (anonymized)		Sent	Completed	Collected	PhD in swarm robotics

Instructions:

Chapter 5

Supplementary Note 5: Technical datasheet

The document below, previously distributed to all human designers, is attached in its original format with contact details redacted for anonymity.

- Technical datasheet of photochromic substance:
  https://findmydata.cloud/uploads/pdf/photochromic-pigment-1.pdf
- Supplier information of photochromic substance:
  https://www.sfxc.co.uk/
- Supplier information of acrylic binder:
  https://www.amazon.com/Amsterdam-Grounds-Acrylic-Binder-1000ml/dp/B01L0PEIGK

Good Life Innovations Ltd
Unit 6, Newhaven Enterprise Centre
Newhaven
BN9 9BA
0207 118 3123
sales@sfxc.co.uk

TECHNICAL DATA SHEET

Product name :	**Purple**
Product code :	**KPMC-12-P**
INCI Name :	Potassium Nitrate-Silica-Sodium Nitrate-Azobenzene （Butyl Methoxydibenzoylmethane ）
CAS No. :	7757.79.1-7631.86.9-7631.99.4-70356.09.1
Date :	07.10.2016

CHARACTERISTICS			RANGE
**Physical properties**			
Characteristics			Purple
Average particle size			1-10 μm
**Chemical composition**			
Potassium Nitrate			50.0-55.0 %
Silica			18.0-23.0 %
Sodium Nitrate			10.0-15.0 %
Azobenzene （Butyl Methoxydibenzoylmethane ）			7.0-22.0 %
**Heavy metals**			
As	< 2 ppm	Hg	< 1 ppm
Ba	< 50 ppm	Ni	< 10 ppm
Cd	< 3 ppm	Pb	<5 ppm
Cr	< 20 ppm	Sb	< 1 ppm
Cu	< 50 ppm	Zn	< 50 ppm
**Microbial purity**			
Total viable Aerobic count			< 100
E. Coli			Absent in 1 g
Pseudomonas aeruginosa			Absent in 1 g
Staphylococcus aureus			Absent in 1 g
Salmonella species			Absent in 1 g
Candida albicans			Absent in 1 g

Please Use Product Name and Product Code When Ordering
