# Supplementary material for: Automatic design of stigmergy-based behaviours for robot swarms
Source: Commun Eng. 2024 Feb 14;3:30. doi: 10.1038/s44172-024-00175-7 (PMC10956014; doi:10.1038/s44172-024-00175-7)
Supplement: Supplementary file 3 — Description of Additional Supplementary Files [file 44172_2024_175_MOESM3_ESM.pdf]

Description of Additional Supplementary Files

File name: Supplementary Video 1

Description: v1-aggregation: This video presents details of the Aggregation mission and demonstrates collective behaviors with physical robots and in simulation for all methods under analysis.

File name: Supplementary Video 2

Description: v2-decision-making: This video presents details of the Decision-Making mission and demonstrates collective behaviors with physical robots and in simulation for all methods under analysis.

File name: Supplementary Video 3

Description: v3-rendezvous-point: This video presents details of the Rendezvous-Point mission and demonstrates collective behaviors with physical robots and in simulation for all methods under analysis.

File name: Supplementary Video 4

Description: v4-stop: This video presents details of the Stop mission and demonstrates collective behaviors with physical robots and in simulation for all methods under analysis.

File name: Supplementary Video 5

Description: v5-robot-view: The video presents an e-puck robot's view of laying and sensing the artificial pheromone.

File name: Supplementary Video 6

Description: v6-experiments: The video presents all real-robots experiment runs performed during this study.
